# Do We Really Need Quantum Mechanics to Describe Plasmonic Properties of Metal Nanostructures?

**DOI:** 10.1021/acsphotonics.2c00761

**Published:** 2022-09-01

**Authors:** Tommaso Giovannini, Luca Bonatti, Piero Lafiosca, Luca Nicoli, Matteo Castagnola, Pablo Grobas Illobre, Stefano Corni, Chiara Cappelli

**Affiliations:** † Scuola Normale Superiore, Piazza dei Cavalieri 7, 56126 Pisa, Italy; ‡ Dipartimento di Scienze Chimiche, Università di Padova, via Marzolo 1, 35131 Padova, Italy; § Istituto di Nanoscienze del Consiglio Nazionale delle Ricerche CNR-NANO, via Campi 213/A, 41125 Modena, Italy

**Keywords:** atomistic, interband, gold, silver, tunneling, field enhancement

## Abstract

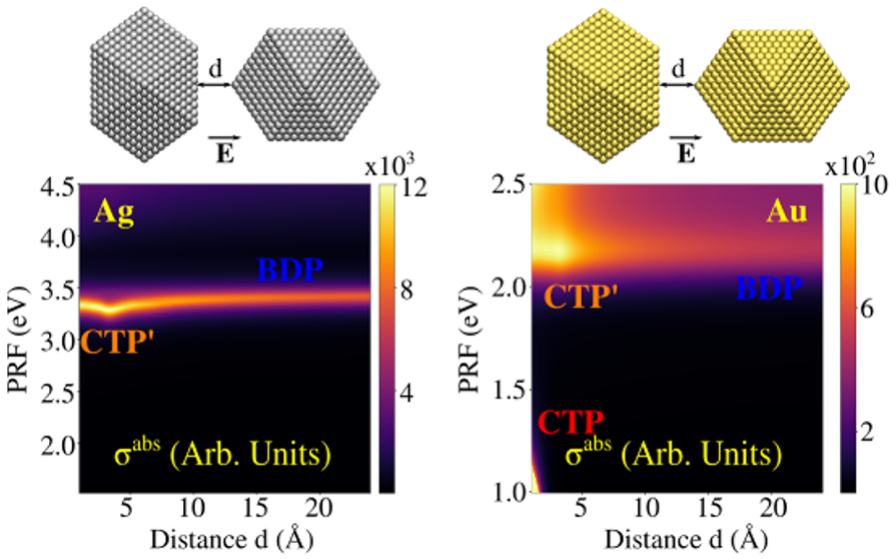

Optical properties of metal nanostructures are the basis of several scientific and technological applications. When the nanostructure characteristic size is of the order of few nm or less, it is generally accepted that only a description that explicitly describes electrons by quantum mechanics can reproduce faithfully its optical response. For example, the plasmon resonance shift upon shrinking the nanostructure size (red-shift for simple metals, blue-shift for *d*-metals such as gold and silver) is universally accepted to originate from the quantum nature of the system. Here we show instead that an atomistic approach based on classical physics, ωFQFμ (frequency dependent fluctuating charges and fluctuating dipoles), is able to reproduce all the typical “quantum” size effects, such as the sign and the magnitude of the plasmon shift, the progressive loss of the plasmon resonance for gold, the atomistically detailed features in the induced electron density, and the non local effects in the nanoparticle response. To support our findings, we compare the ωFQFμ results for Ag and Au with literature time-dependent DFT simulations, showing the capability of fully classical physics to reproduce these TDDFT results. Only electron tunneling between nanostructures emerges as a genuine quantum mechanical effect, that we had to include in the model by an ad hoc term.

## 1 Introduction

The recent progress in nanoscience has allowed to experimentally reach the atomistic detail in the geometrical arrangement of metal nanoaggregates.^1−5^ This has paved the way for many technological applications, including the creation of local hot-spots featuring enormously enhanced electric field, that has allowed single molecule detection, and even submolecular resolutions, when coupled to surface enhanced spectral techniques.^6−13^ A deep understanding of the peculiarities of these structures may benefit of an unavoidable interplaying between theory and experiments. At subnanometric scales such as for atomistically defined needles/tips, quantum effects play an important role in the plasmonic response and therefore need to be considered.^14−23^ As a result, a unified theoretical approach to describe the plasmonic properties of metal nanoaggregates under different regimes needs to consistently take into account the physical phenomena underlying the quantum and classical response.^19,24,25^

Large-size nanoparticles are typically described by means of classical electrodynamics, such as the Mie theory,^26^ the Discrete Dipole Approximation (DDA),^27^ the electromagnetic Finite Difference Time Domain (FDTD),^28^ or the Boundary Element Method (BEM).^29−34^

Nonlocal corrections can be considered by exploiting spatially dependent dielectric function based models^35^ or hydrodynamic models,^36−38^ which are also able to account for the electron spill-out effect that determines the near-field generated in plasmonic hot-spots of *d*-metals.^39−41^ However, these models substantially discard atomistic details, which could become relevant when studying surface-assisted spectroscopic properties.^42^ It is also worth noting that electron spill-out and nonlocal effects can effectively be treated by means of surface response functions, which would need to be specified for different surface planes.^43,44^ Limitations of these approaches for nanoparticles have recently been discussed.^45^ In this context, ab initio modeling, at the Time-Dependent Density Functional Theory (TDDFT) level, is still considered the most accurate approach to deal with these effects;^46−50^ however, it can only treat relatively small metal nanoparticles (NP; with diameter < 5 nm); therefore, real-size systems cannot be afforded due to the prohibitively large computational cost. Such a situation naturally leads to the conclusion that an explicit quantum mechanical treatment of electrons, such as DFT and TDDFT, is mandatory to provide a realistic picture of plasmonic phenomena in this size regime. Such a conclusion is not really challenged by the existing classical atomistic approaches to nanoplasmonics,^51−57^ that, while delivering accurate results, are based on fitting very general classical response expressions on TDDFT calculations, retaining therefore the physical basis of the latter.

In this paper, we explore whether a classical atomistic method based on essentially classical ingredients (Drude conduction mechanism and classical polarizabilities to reproduce interband polarization) can reproduce the optical response of complex plasmonic nanostructures. To this goal, we propose a physically robust approach to describe the plasmonic properties of sizable metal nanoaggregates characterized by the presence of interband transitions. Together with collective electronic excitations, they determine the plasmonic response of noble metal nanoparticles. The model is based on the recently developed ωFQ method,^32,58−61^ in which each metal atom is endowed with a net charge, which varies as a response of the external electric field. The charge-exchange between atoms is governed by the Drude mechanism. As an element of mere quantum mechanical origin that we found essential to add to our classical atomistic picture, the Drude charge exchange is modulated by quantum tunneling, which guarantees a correct description of the optical response for subnanometric junctions.^15,16,18,20,22,58,62,63^ Although the method has been successfully applied to sodium nanostructures and graphene-based materials,^58,59^ the basic formulation of ωFQ overlooks interband contributions, thus, it is unsuitable to describe the plasmonic properties of nanostructures based on *d*-metals.^64−68^ Here, we substantially extend ωFQ so to assign each metal atom with an atomic complex-valued polarizability (i.e., a complex-valued dipole moment), appropriately tuned to model interband effects. The resulting approach is called ωFQFμ (frequency-dependent fluctuating charges and fluctuating dipoles) by analogy with a parent polarizable approach, which has been proposed by some of the present authors to treat completely different chemical systems.^69−71^ The theoretical basis of the approach stems from the evidence that *d*-states can efficiently be treated as polarizable shells placed at lattice positions.^67^

Notice that ωFQFμ was developed from a different perspective compared to other classical atomistic approaches.^51−54^ Indeed, ωFQFμ is built from textbook bulk metal physics rather than fitting of generic polarizability and capacity frequency-dependent expressions. This provides practical benefits as well. In fact, Drude-tunneling and interband regimes are perfectly decoupled in the two terms that depend on ωFQs^58^ and ωFμs, thus allowing for a fine, physically guided, tuning of the plasmonic response. In the following, we show that ωFQFμ is able to correctly reproduce the plasmonic properties of Ag and Au nanostructures as a function of size and shape, and also their plasmonic response when forming subnanometer junctions. Remarkably, the favorable scaling of the method permits to afford large systems (more than 10^4^ atoms), which cannot be treated at the quantum-mechanical level. Also, the ability of ωFQFμ to fully retain the atomistic detail is crucial to reproduce not only the plasmonic response but also near-field enhancements, which play a key-role in near-field enhanced spectroscopies.^72,73^

## 2 Theoretical Model

ωFQFμ is a fully atomistic, classical approach which substantially extends ωFQ, which assigns to each metal atom a time dependent charge. Under the action of a time dependent external electric field, metal atoms exchange charge via the Drude conduction mechanism, which is further assisted by quantum tunneling, which limits the charge transfer among nearest neighboring atoms and makes the interaction decrease with the typical exponential decay.^58^ In particular, ωFQ charge equation of motion in the frequency domain (ω) reads:^58^
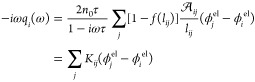
1where *q*_*i*_(ω), a complex-valued quantity, is the Fourier component at the frequency ω of the oscillating atomic charge on atom *i*. *n*_0_ is the metal density, τ the friction time,  is the effective area connecting *i*th and *j*th atoms, and *l*_*ij*_ is their distance. ϕ^el^ is the electrochemical potential acting on each metal atom, which takes into account the interactions between the different atoms and their interaction with the external electric field, which oscillates at frequency ω. *f*(*l*_*ij*_) is a Fermi-like function mimicking quantum tunneling:^58^
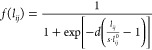
2where *l*_*ij*_^0^ is the equilibrium atom–atom distance, whereas *d* and *s* are dimensionless parameters that determine the sharpness and the center of the Fermi function *f*(*l*_*ij*_), respectively. Their values can be determined by comparing computed results with reference ab initio data (see also Section S1 in the Supporting Information (SI)). In eq 1, Drude and tunneling terms are collected in the **K** matrix. The parameters entering ωFQ all have a clear microscopic physical meaning. Therefore, their values can be either chosen by an independent experiment or fitted to reproduce higher level results, and then the soundness of their values can be judged. We took the latter perspective, as discussed in the SI.

Due to its physical foundations, ωFQ cannot describe the specificity of metals featuring *d*-electrons, which contribute to interband transitions and that substantially affect the plasmon response.^64−68^ Therefore, we here extend ωFQ into a novel method, ωFQFμ, in which each atom is assigned a charge and an additional source of polarization, that is, an atomic polarizability (to which an induced dipole moment is associated). The presence of the dipole moments is included in eq 1 by taking into account the interaction between charges and dipoles in the electrochemical potential. The induced dipole moments **μ**_*i*_ are instead obtained by solving the following set of linear equations:
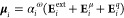
3where, **E**^ext^, **E**^μ^, and **E**^*q*^ are the external electric field and those generated by the other dipole moments and charges, respectively. α^ω^ is the atomic complex polarizability, which is introduced to describe interband transitions. Remarkably, α^ω^ can easily be obtained by extracting interband contributions from the experimental permittivity function (see Section S1 in the SI), with no need to introduce a posteriori adjustable parameters.

To effectively couple charges and dipoles, that is, to simultaneously account for Drude and interband transitions, eqs 1 and 3 need to be solved simultaneously. By explicitly indicating all terms, the problem can be recast as the following set of linear equations:
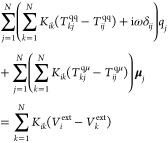
4
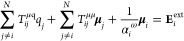
5where *T*^qq^, *T*^qμ^, and *T*^μμ^ define charge–charge, charge–dipole, dipole–dipole interactions, respectively. By imposing *T*^μμ^ diagonal elements to correspond to 1/α^ω^, eqs 4 and 5 can be written in a compact matrix formulation as
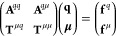
6where the complex **A** matrices include interaction kernels and the *K*_*ij*_ terms (see Section S1 in the SI for more details). The right-hand side of eq 6 accounts for external polarization sources, that is, the electric potential and field calculated at atomic positions:
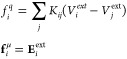
Notably, any kind of plasmonic materials can be modeled by ωFQFμ because it integrates all relevant physical ingredients (i.e., Drude conduction, electrostatics, quantum tunneling and interband transition) via eq 1 and eq 3. Also, once complex-valued charges and dipoles are computed, the absorption cross section σ^abs^ and the induced electric field can easily be calculated (see Section S1 in the SI for more details).

To conclude this section, it is worth noting that other atomistic, classical models, belonging to the Discrete Interaction Model (DIM) class, have been proposed to describe the plasmonic response of noble-metal nanoparticles.^54,74^ Among them, the coordination-dependent–Discrete Interaction Model (cd-DIM)^74^ is able to properly describe the size dependency of the Plasmon Resonance Frequency (PRF) for Ag NPs and the plasmonic response of Ag dimers, which is governed by quantum tunneling. Within this model, both effects arise from a modification of the coordination of surface atoms and the consequent modification of the atomic polarizability that is considered to be a parametrized function of the coordination number. In our approach the first effect genuinely originates from the screening effect of *d*-electrons, which is physically modeled by the presence of the α^ω^ term. A correct description of the plasmonic response of dimers arises from the phenomenological introduction of the quantum tunneling via the Fermi function in eq 2.

## 3 Results and Discussion

### 3.1 Optical Response of Metal Nanoparticles: ωFQFμ versus TDDFT Results

Here the capability of ωFQFμ at describing typical plasmonic response properties of single metal nanoparticles is discussed.^75^ Although the model is completely general, it is here applied to Ag and Au nanoparticles (NP), for which α^ω^ values are obtained from the permittivity functions reported by Etchegoin et al. in ref (76) and fitted in ref (77) (see also Section S1 in the SI). For both metals, we first consider NPs with three different geometrical arrangements, namely, truncated cuboctahedron (cTO), icosahedral (Ih), and ino-decahedron (i-Dh), which are all characterized by atomistically defined edges.^54^ Their plasmonic properties are studied as a function of the size (from a minimum of 85 atoms, ∼5 Å radius, to a maximum of 43287 atoms, ∼65 Å radius).^78−80^ Note that geometry relaxation is not considered, because it only slightly affects optical responses.^23,81−86^

**Figure 1:**
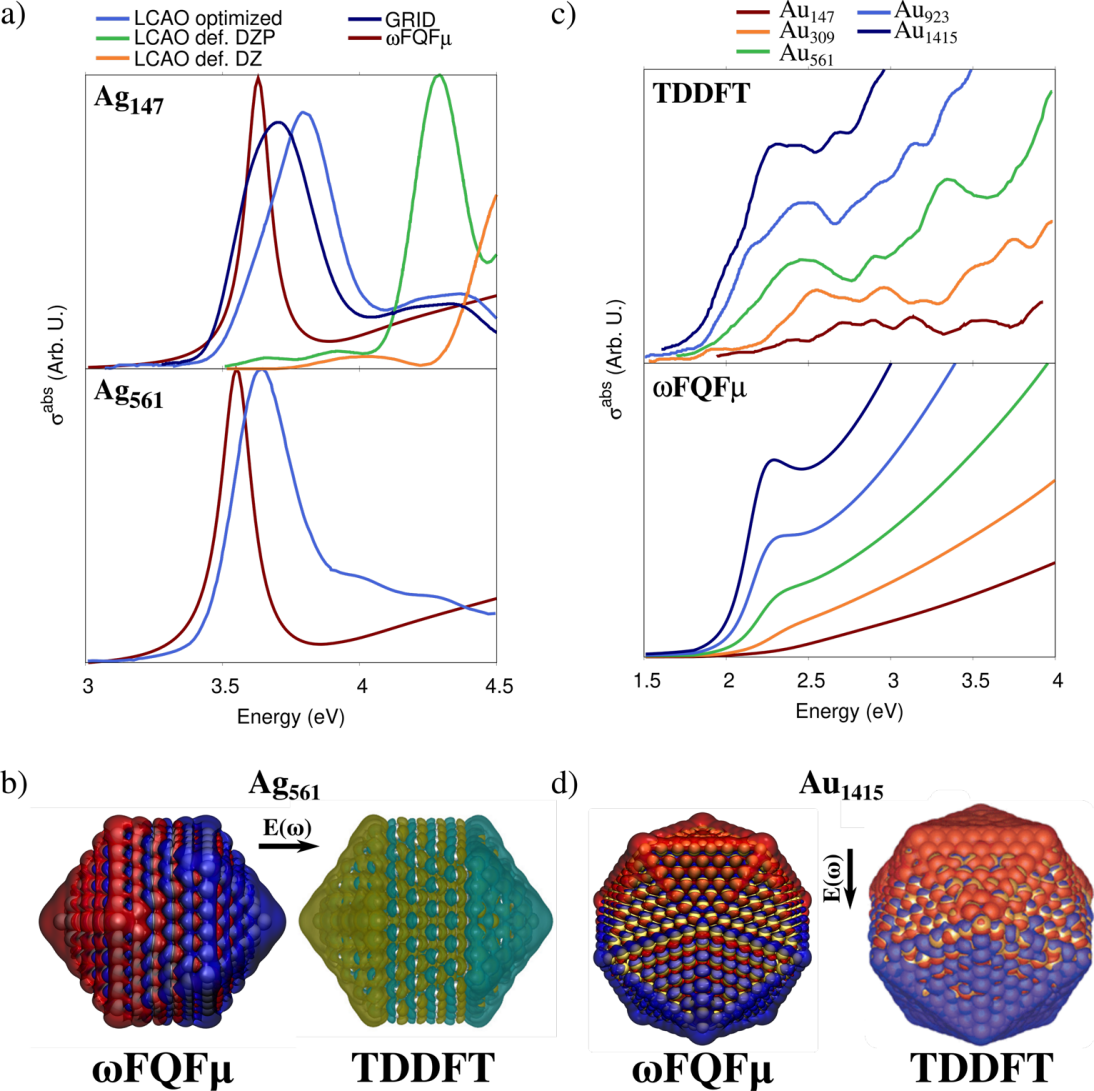
(a) Computed ωFQFμ and TDDFT σ^abs^ for Ag_147_ (∼0.8 nm radius) and Ag_561_ (∼1.4 nm radius). TDDFT results are reproduced from ref (87) and obtained by exploiting different basis sets (LCAO optimized, def. DZ (double-ζ), def. DZP (double-ζ polarized)) and on a real-space grid (GRID). (b) ωFQFμ and TDDFT^87^ plasmon densities for Ag_561_. TDDFT plasmon densities adapted with permission from ref (87). Copyright 2015 APS Publications. (c) ωFQFμ and TDDFT^88^ σ^abs^ for Au_147_–Au_1415_ (∼1.9 nm radius). (d) ωFQFμ and TDDFT^88^ plasmon densities for Au_1415_. TDDFT plasmon densities adapted with permission from ref (88). Copyright 2014 ACS Publications. ωFQFμ isovalues are set to 0.002 and 0.0005 au for Ag and Au, respectively.

Although sizable NPs cannot be afforded by ab initio methods, they can indeed be treated by ωFQFμ,^60^ at a reasonable computational time (on average, 59 min on Intel(R) Xeon(R) Gold 5120 CPU @ 2.20 GHz, 28 processors, for each frequency given in input for the structure composed of 43287 atoms). As an example of the performance of ωFQFμ, Kuisma et al.^87^ have reported that the calculation of the optical spectrum of Ag_561_ (∼1.4 nm radius) in Ih geometry with the time-propagation (TP) approach to TDDFT requires a wall time of 42.0 h with 512 cores.^87^ For the same system, ωFQFμ only requires 25 s on the aforementioned platform. Remarkably, ωFQFμ and reference TDDFT data are very similar (see Figure 1a, bottom panel). Slight discrepancies in the PRF and band broadening among the two models can be justified by considering that TDDFT results substantially vary as a function of the basis set; this is demonstrated by the data shown in Figure 1a (top panel), which are taken from ref (87). Clearly, reference ab initio data display large variability of almost 1 eV when moving from linear combinations of atomic orbitals (LCAO) with different basis sets to real space grid calculation (GRID) results. We also remark that the width of peaks is chosen arbitrarily in TDDFT calculations.

A similar comparison can be performed for Au Ih NPs. TDDFT absorption cross sections reproduced from ref (88) and calculated at the ωFQFμ level are reported in Figure 1c,d. Also, in this case, ωFQFμ can correctly reproduce PRF trends as a function of the NP size and the relative intensities of the bands for the different NPs.

One of the most peculiar features of noble metal NPs and, in general, of *d*-metals is that the PRF blue shifts as the size of the system decreases, in contrast to what happens for simple metals (and correctly reproduced by ωFQ^58^). The physical origin of this blue-shift has been studied in the past.^67^ The screening of the Coulomb interaction among conduction electrons (that determines the plasmon frequency) by the localized *d*-electron core interplays with conduction electrons spill-out at the cluster surface. In short, the *d*-electron core screens electron–electron repulsion, thus decreasing the *d*-metal plasmon frequency compared to what is expected on the basis of the free-electron density of the metal. At the surface, conduction electrons spill out of the structure; in ωFQ and ωFQFμ, the finite size of each atomic distribution accounts for this effect. In parallel, the *d*-electron core, which is localized on the metal atom (described by a point dipole in ωFQFμ) cannot effectively screen the electron–electron repulsion. As a result, the plasmon frequency moves back to the nonscreened, free-electron value. Remarkably, ωFQFμ can indeed describe this mechanism because it provides the right result for the right reason. In fact, if the *d*-electron core response is artificially switched-off (i.e., α^ω^ → 0 in eq 3), the plasmon frequency (which is overall increased), red shifts for metal nanoparticles, as it is expected based on spill-out effects only.^58,82^ This is demonstrated by the plots reported in Figure S3 in the SI.

**Figure 2:**
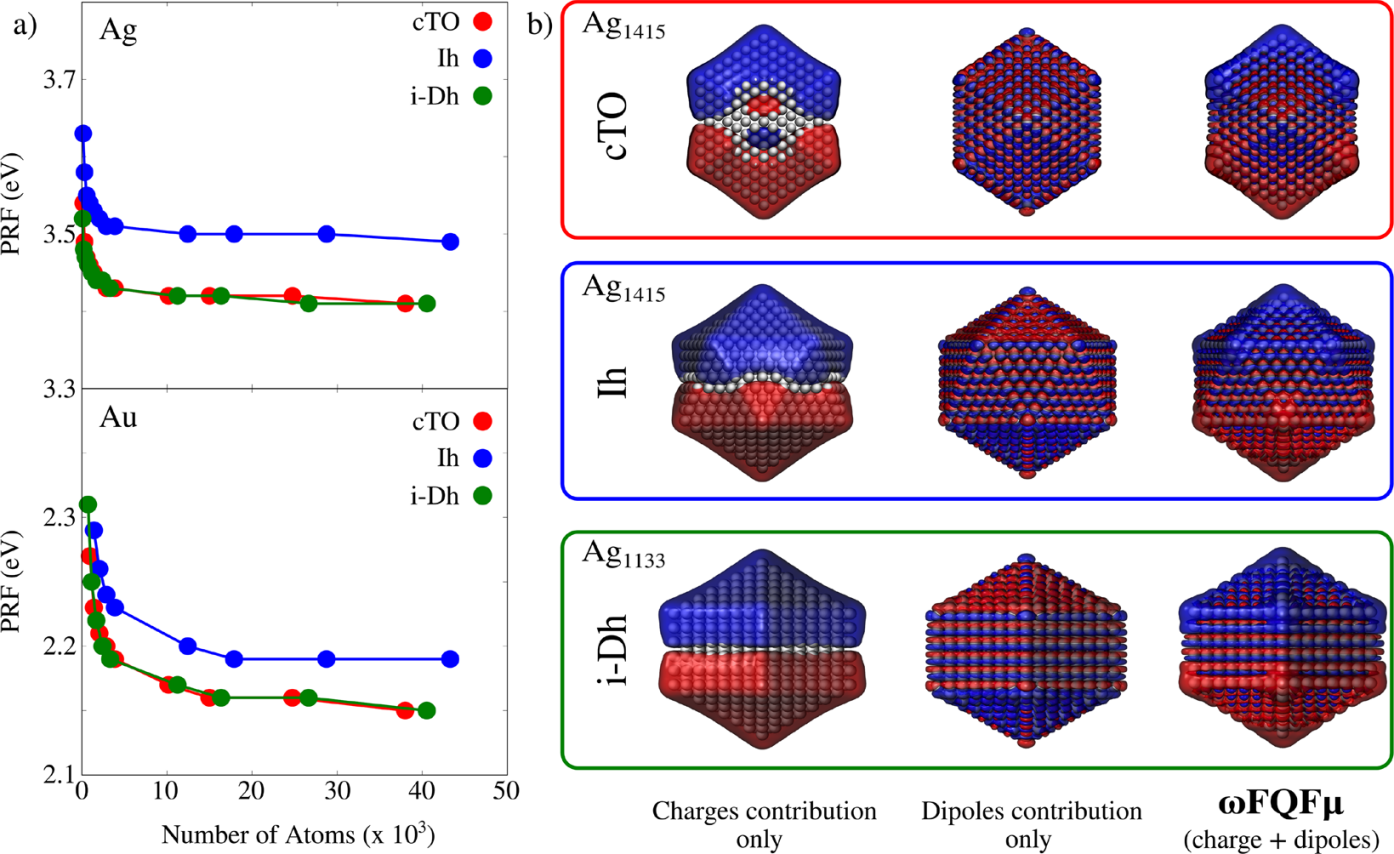
(a) Ag and Au PRF for cTO, Ih, and i-Dh NPs as a function of the number of atoms. (b) Ag densities calculated at the PRF for cTO (top), Ih (middle), and i-Dh (bottom) geometries. Charge and dipole contributions are plotted together with ωFQFμ plasmon densities. Isovalues are 0.002 and 0.0005 au for Ag and Au, respectively.

The dependence of computed PRFs on the number of atoms is reported in Figure 2a for different geometries; the plots clearly demonstrate that ωFQFμ can indeed correctly reproduce the previously reported trends. Also, the linear fit of Ag Ih PRFs as a function of the inverse of the NP diameter permits to linearly extrapolate PRF = 3.47 eV for an infinite diameter. This value is in almost perfect agreement with the mesoscopic limit of 3.43 eV, as obtained at the quasi-static FDTD (QSFDTD) level,^87^ and in excellent agreement with the extrapolated ab initio value of 3.35 eV (see also Figure S4 in the SI).^87^

The investigation of plasmon densities (i.e., the imaginary part of the charge density induced by a monochromatic electromagnetic field oscillating at the PRF) is of fundamental importance for correctly characterizing the plasmon resonance. Computed densities at the PRF for the largest structures in each geometrical arrangement are depicted in Figure 2b. In all cases, they represent a dipolar plasmon. Our result can be compared with reference ab initio data for Ag_561_ (∼1.4 nm radius, see Figure 1b) and Au_1415_ (∼1.9 nm radius, see Figure 1d). In both cases, the agreement is almost perfect.

For the purpose of a deeper theoretical analysis, ωFQFμ can also be used to decouple the contributions of Drude and interband transitions to the total plasmon density. Charge and dipole contributions are graphically depicted in Figure 2b for selected Ag NPs (see Figure S5 in the SI for Au NPS). Clearly, the two plasmon densities are associated with dipole moments in opposite directions. This finding remarks the screening role of the *d*-electrons response.^41,67^ As explained above, this results in the typical blue shift observed for *d*-metals.

**Figure 3:**
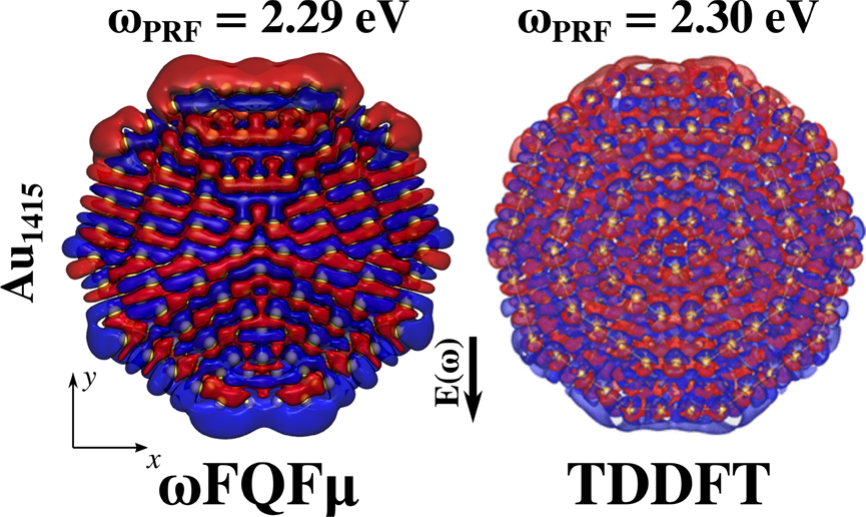
ωFQFμ and TDDFT^88^ Au_1415_ (∼1.9 nm radius) plasmon densities in the central region of the cluster (−2 < *z* < 2 Å). TDDFT plasmon densities adapted with permission from ref (88). Copyright 2014 ACS Publications.

To further demonstrate the ability of ωFQFμ to correctly take into account screening effects in *d*-metal NPs, we can compare density distributions in inner regions. Computed ωFQFμ and TDDFT densities for Au_1415_ Ih NP (∼1.9 nm radius), in the central region of the cluster (defined for −2 < *z* < 2 Å), are depicted in Figure 3. Notice that densities are computed at the corresponding PRFs, which only differ by 0.01 eV. Coherently with the results reported in Figure 1d, positive and negative charges are located at the top and bottom surface regions, respectively. However, ωFQFμ and TDDFT densities are mainly characterized by a local charge distribution around each Au atom, which is polarized along an opposite direction with respect to the polarization of the surface density. Such a behavior is related to screening effects, which are correctly taken into account by our atomistic, yet classical model.

**Figure 4:**
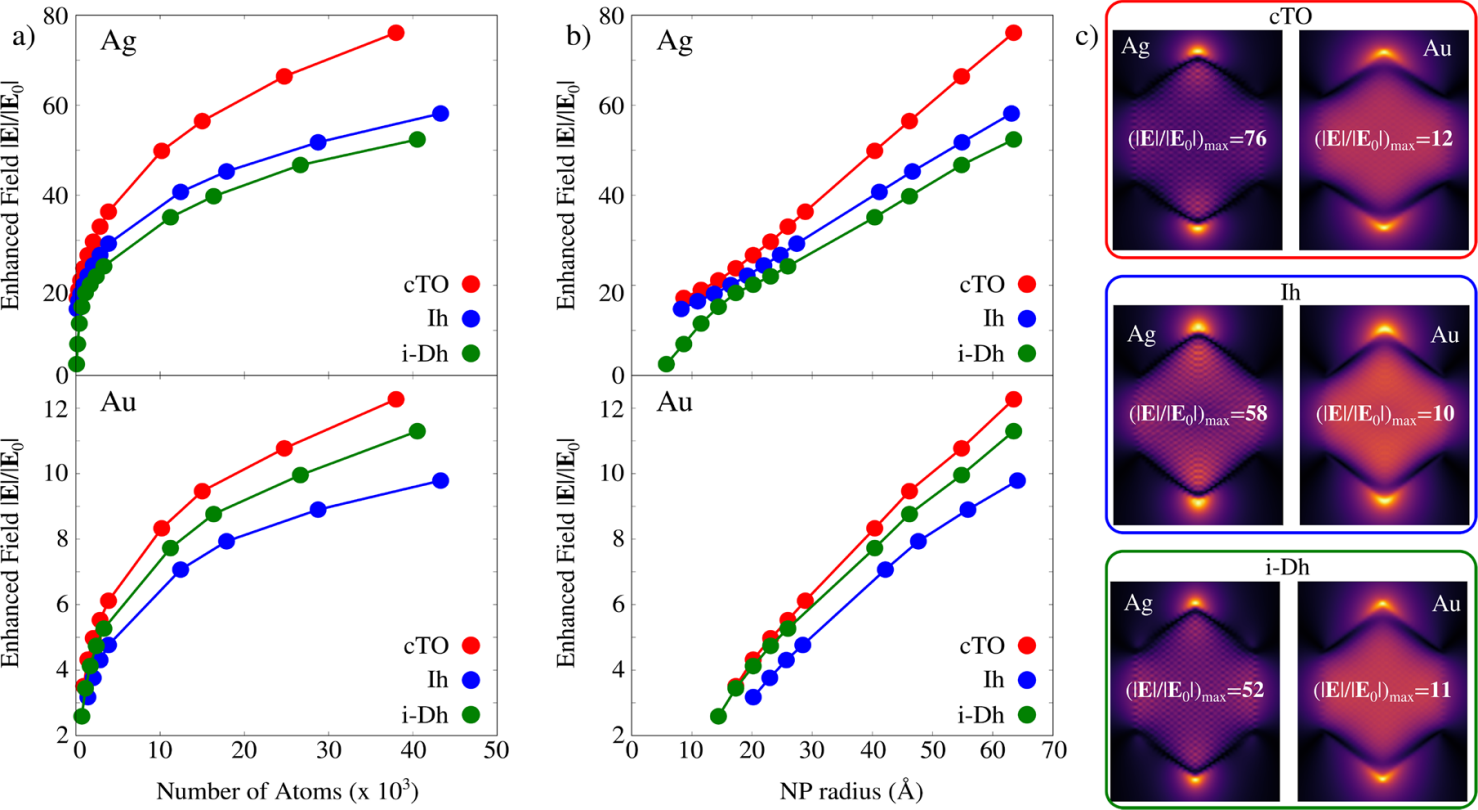
Ag and Au enhanced electric field |**E**|/|**E**_0_| calculated at 3 Å from the tips for cTO, Ih, and i-Dh NPs as a function of the number of atoms (a) and NP radius (b). (c) Ag and Au |**E**|/|**E**_0_| color maps for cTO (top), Ih (middle), and i-Dh (bottom) geometries.

In Figure 4a,b the total enhanced electric field (|**E**|/|**E**_0_|, where **E**_0_ is the external electric field intensity) at the PRF is reported. Such quantity (elevated to the fourth power) is related to field enhancement factors that are measured in SERS experiments.^10^ The dependence of |**E**|/|**E**_0_| factors as a function of the number of atoms and the NPs radius is reported in Figure 4a and b, respectively. Notice that enhancement factors are computed at a distance of 3 Å from the tip of each structure; according to many previous reports, it corresponds to the typical adsorption distance of molecular systems.^89^ |**E**|/|**E**_0_| color maps at the PRF for each geometrical arrangement are graphically displayed in Figures 4c. As expected, |**E**|/|**E**_0_| maximum values correspond to tips, and are reported for cTO geometries (for both gold and silver), where edges are the sharpest. Interestingly, for all arrangements, |**E**|/|**E**_0_| follows a  trend (*N* being the number of atoms) and, thus, a linear trend with respect to NP radius, because the difference in the electric potential linearly increases with the NP intrinsic size. Remarkably, ωFQFμ is also able to quantify the differences between Ag and Au NPs, being the latter associated with much lower enhancement factors as compared to the former, as expected in this frequency range.

As a final comment, it is remarkable that ωFQFμ is able to describe nonlocal effects. To demonstrate that, we artificially changed the total electric potential and field acting on a specific atom placed at the surface or at the centroid of Ag_147_ (∼0.8 nm radius) and Ag_3871_ (∼2.7 nm radius) structures in the Ih configuration. As a result, independently of the atom position, not only charge and the dipole of the perturbed atom are modified, but also those of other atoms (see Figure S6 in the SI).

### 3.2 Subnanometer Junctions

**Figure 5:**
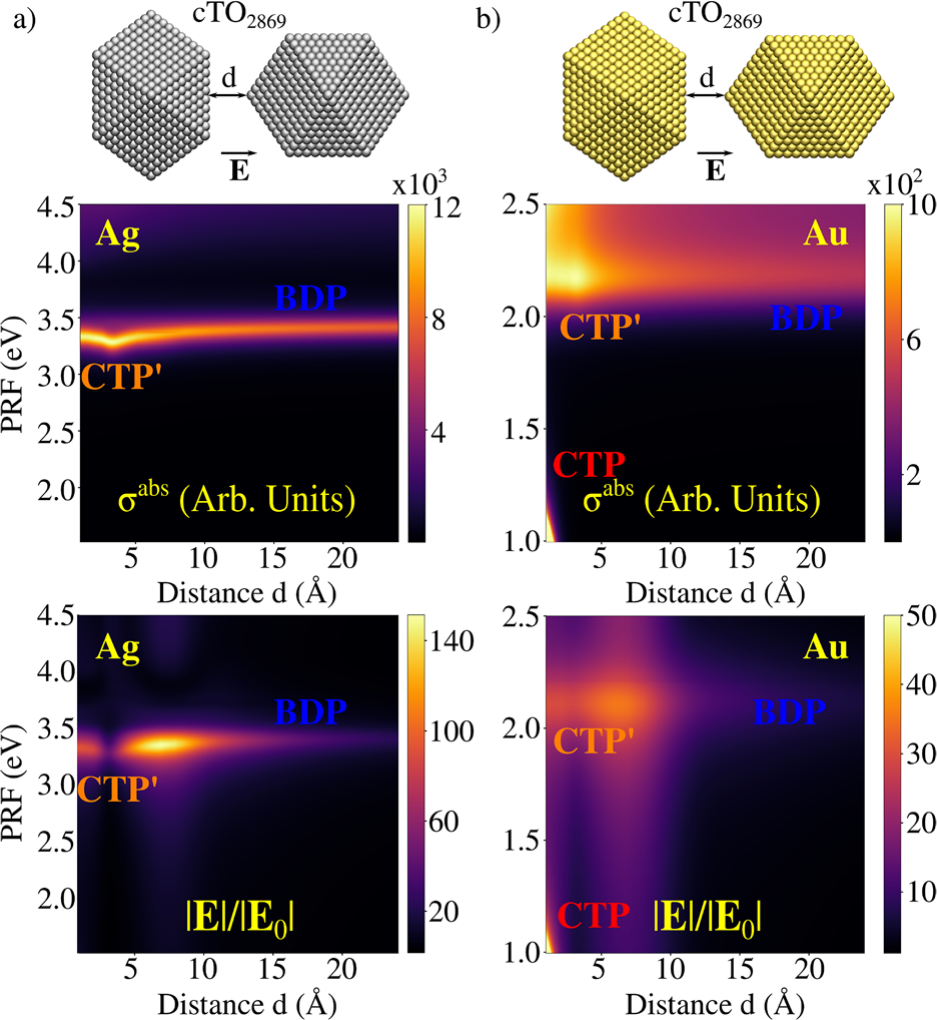
Ag (a) and Au (b) cTO_2869_ dimers in surface–tip geometrical arrangement. Color maps of σ^abs^ and |**E**|/|**E**_0_| as a function of the PRF and the distance between the two NPs are reported in the middle and bottom panels, respectively.

**Figure 6:**
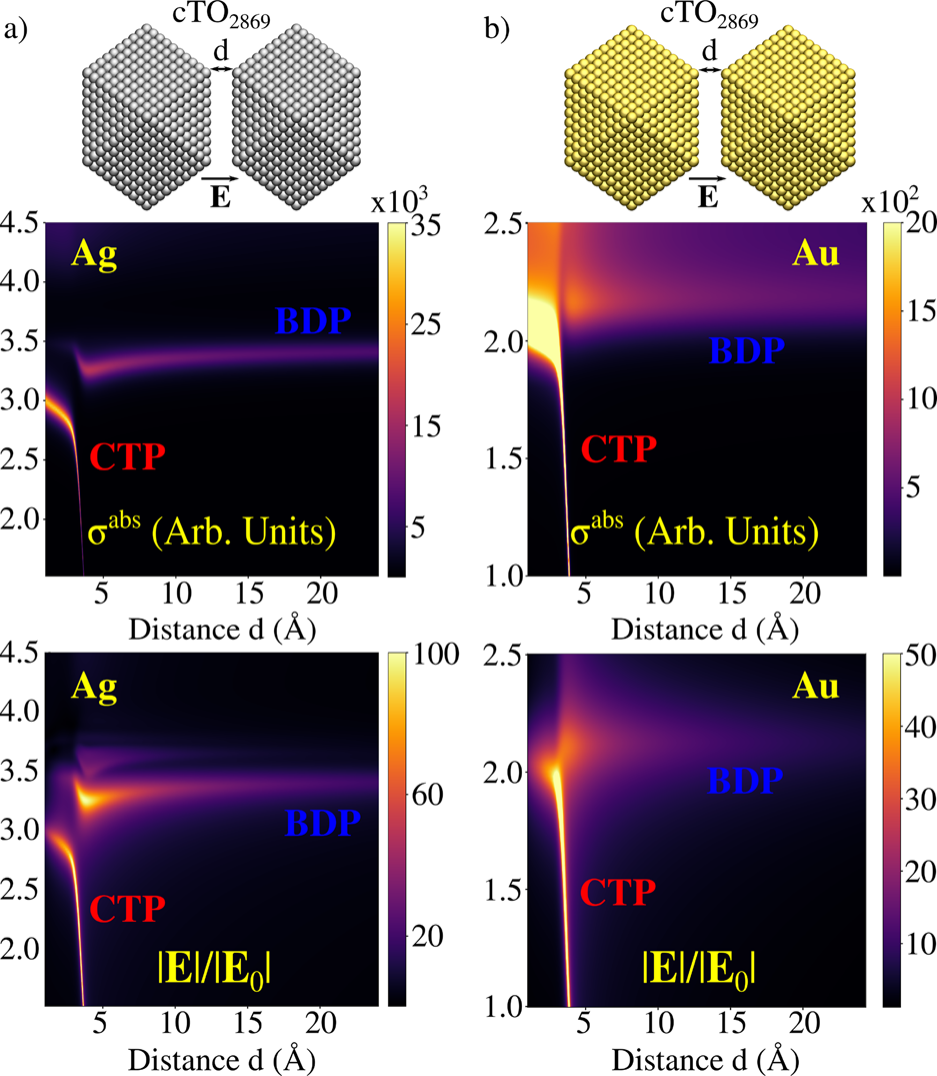
Ag (a) and Au (b) cTO_2869_ dimers in surface–surface geometrical arrangement. Color maps of σ^abs^ and |**E**|/|**E**_0_| as a function of the PRF and the distance between the two NPs are reported in middle and bottom panels, respectively.

In this section we will show that ωFQFμ has the potential to describe hot-spots in subnanometer junctions in a physically consistent manner. This is possible due to the account for quantum tunneling (eq 1), which dominates the plasmon response in these systems. To showcase ωFQFμ performances, we study Ag_2869_/Au_2869_ (∼2.6 nm radius) cTO dimers. In particular, we select two different morphologies obtained by approaching two NPs so to obtain surface–tip or surface–surface geometrical arrangements (see Figures 5 and 6).^90^ Note that for both structures the structural relaxation effects are not taken into account, similarly to previous studies.^81^ However, to study the effects of structural relaxation we have modeled surface roughness as derived from an atomic adjustment on the surface of one of the two cTO NPs in the surface–surface arrangement (see Figure S7 in the SI).

For the ideal cTO dimers, we investigate both σ^abs^ and |**E**|/|**E**_0_| calculated at the gap’s center as a function of the distance *d* between the two monomers, in the range 1–24 Å. Computed spectra (Figure 5a,b) are characterized by a high energy peak (∼3.5 eV for Ag and ∼2.2 eV for Au), which red-shifts as *d* decreases. However, a clear discontinuity occurs at around 4 Å, where quantum tunneling plays a relevant role; in fact, such a distance is close to the Ag–Ag and Au–Au equilibrium distances.

For Au dimers, a second peak at lower energies is visible (*d* < 2 Å, PRF < 1.5 eV), which blue-shifts as *d* decreases (see Figure 5b). This band is not present for Ag, because its PRF falls below the investigated frequency range. Highest energy PRF corresponds to the typical Boundary Dipolar Plasmon, BDP, whereas the low-energy peak is associated with a Charge Transfer Plasmon, CTP, where a dipole moment arises in the whole structure (see Figures S8 and S9 in the SI). Note that for distances at which the CT mechanism takes place (for *d* < 4 Å), the high energy peak is associated with a high-order charge transfer plasmon, usually called CTP′. The clear discontinuity highlighted in σ^abs^ plots is also evident in the case of the computed |**E**|/|**E**_0_| values, which are reported as a color map as a function of the distance in Figure 5, bottom panels. For Ag, the maximum |**E**|/|**E**_0_| is depicted before the two NPs enter in the CT regime, whereas the opposite holds for the Au dimer, for which it corresponds to the CTP peak.

The surface–surface arrangements show similar plasmonic features, independently of the metal nature (see Figure 6a,b). In fact, both spectra are characterized by the presence of two bands when *d* < 4 Å: a CTP and a CTP′ peak (very low in intensity for Ag and fused with CTP for Au), which blue-shift or red-shift as *d* decreases, respectively. For *d* > 4 Å spectra are instead dominated by the BDP mode. The associated |**E**|/|**E**_0_| factors are plotted as a function of the distance in Figure 6, bottom panels. The computed values are of the same order of magnitude as for the previous case. Also, the maximum enhancement for Ag is shown at about 3.3 eV, for a distance of about 4 Å, that is, when CT effects start to dominate the plasmonic response. For Au, the maximum |**E**|/|**E**_0_| occurs at lower distances (*d* ∼ 3 Å), however it is associated with the CTP′ plasmonic mode, differently from the tip–substate arrangement. Interestingly, in case of Ag, a clear additional region of enhancement is displayed at energies larger than 3.5 eV, for *d* > 4 Å. This region is associated with a high-order plasmon, which is indeed dark in the σ^abs^.

Finally, note that, for both studied geometries, the maximum |**E**|/|**E**_0_| is 3–5 times larger than the corresponding factor obtained for the Ag monomer and even 10 times for Au.

## 4 Summary and Conclusions

We have discussed the prediction of a classical physics based model for nanoplasmonics, ωFQFμ. ωFQFμ is a general atomistic approach to describe the plasmonic features of complex metal nanostructures characterized by interband transitions. In the model, which is formulated in the frequency domain, each atom of the structure is endowed with both a complex-valued charge and dipole, which are determined by solving response equations to an external monochromatic electric field. The two polarization sources conceptually describe the two fundamental mechanisms occurring in *d*-metals, that is, Drude conduction (charges) and *d*-electrons polarization (dipoles). Remarkably, charges and dipoles mutually interact; therefore, all physical features of metal nanostructures are considered. ωFQFμ is fully classical, therefore nanostructures of realistic size can be computed with accuracy comparable to full ab initio calculations, but with enormously lower computational cost. Note that the interband polarizability in eq 3 arises from the quantum nature of the system; however, in our approach it is modeled without explicitly considering the quantum nature of the constituting atoms.

From a conceptual point of view, demonstrating that ωFQFμ is able to reproduce the findings of a fully quantum description of the system is clearly questioning the notion that an explicit quantum mechanical treatment is needed to describe the change in plasmonic properties upon shrinking of the nanostructure size. From the results presented in this work, it turns out that only quantum tunneling (relevant for nanoaggregates and nanojunction) is inaccessible by such classical physics, and we had in fact to phenomenologically include the tunneling in ωFQFμ. It is finally worth noting that another relevant quantum effect, Landau damping, is not included in this classical modeling and would in principle require adding a phenomenological correction to ωFQFμ.

From a computational perspective, the developed method paves the way for an investigation of the plasmonic properties of realistic metal nanoparticles, characterized by complex shapes that require an atomistic detail.

## Supporting Information Available

Details on ωFQFμ equations and parametrization; Computational details; Ag and Au single NPs absorption cross sections and comparison with reference data; Demonstration of ωFQFμ nonlocality; Plasmon densities for Ag/Au cTO_2869_ dimers (PDF)
