# Supplementary material for: Do We Really Need Quantum Mechanics to Describe Plasmonic Properties of Metal Nanostructures?
Source: ACS Photonics. 2022 Sep 1;9(9):3025–34. doi: 10.1021/acsphotonics.2c00761 (PMC9502030; doi:10.1021/acsphotonics.2c00761)
Supplement: Supplementary file 1 — ph2c00761_si_001.pdf [file ph2c00761_si_001.pdf]

Supporting Information for: Do We Really Need Quantum Mechanics to Describe Plasmonic Properties of Metal Nanostructures?

Tommaso Giovannini,[*,†] Luca Bonatti,[†] Piero Lafiosca,[†] Luca Nicoli,[†] Matteo Castagnola,[†] Pablo Grobas Illobre,[†] Stefano Corni,[‡,¶] and Chiara Cappelli[*,†]

†*Scuola Normale Superiore, Piazza dei Cavalieri 7, 56126 Pisa, Italy.*

‡*Department of Chemical Sciences, University of Padova, via Marzolo 1, 35131 Padova, Italy*

¶*CNR Institute of Nanoscience, via Campi 213/A, 41125 Modena, Italy*

E-mail: tommaso.giovannini@sns.it; chiara.cappelli@sns.it

S1 Theoretical Model

The model we are introducing in this paper, ωFQFμ, has its fundamentals on the Fluctuating Charges Fluctuating Dipoles (FQFμ) force field, which is usually adopted for describing molecular systems,[1–3] and it is the extension of ωFQ to the case of d-metals. In particular, each atom is endowed with a complex electric charge, whose value is regulated by the Drude theory of conduction electrons, and a complex dipole moment which models the polarizability of d-electrons. Under the action of an external monochromatic electric field $\mathbf{E}(\omega)$, the imaginary part of both charges and dipoles determines the absorption spectrum of a metal nanoparticle.

We start our derivation by writing the interaction energy of a system constituted by both charges and dipoles. Therefore, the energy must include charge-charge, charge-dipole and dipole-dipole energy terms. In addition, if the system is subject to an external electric field, charge-external field and dipole-external field interactions need to be included. The energy and the chemical potential then read:

$$U = \chi^T \cdot \mathbf{q} + \frac{1}{2}\mathbf{q}^T T^{qq}\mathbf{q} + \frac{1}{2}\boldsymbol{\mu}^T \boldsymbol{T}^{\mu\mu}\boldsymbol{\mu} - \mathbf{q}^T \boldsymbol{T}^{q\mu}\boldsymbol{\mu} + \mathbf{q}^T \cdot \mathbf{V}^{ext} - \boldsymbol{\mu}^T \cdot \mathbf{E}^{ext} \tag{S1}$$

$$\mu_i^{el} = \frac{\partial U}{\partial q_i} = \sum_j T_{ij}^{qq} q_j - \sum_j \boldsymbol{T}_{ij}^{q\mu} \boldsymbol{\mu}_j + \chi_i + V_i^{ext} \tag{S2}$$

where $\boldsymbol{\mu}$ is a vector containing all the dipoles of the atoms in the structure and the tensors $\boldsymbol{T}$ are the kernels for charge and dipole interactions, whose subscript indicates the considered interaction. In particular, the diagonal elements of T^{qq} and $T^{\mu\mu}$ are the chemical hardnesses $\boldsymbol{\eta}$ and the inverse of the static atomic polarizability $\boldsymbol{\alpha}^{\mathbf{0}}$:

$$T_{ii}^{qq} = \eta_i \tag{S3}$$

$$T_{ii}^{\mu\mu} = \frac{1}{\alpha_i^0} \tag{S4}$$

In $\omega FQF\mu$ both charges and dipoles are described by Gaussian functions:[1,4]

$$q_i \rho_{q_i}(\mathbf{r}) = \frac{q_i}{\pi^{3/2} R_{q_i}^3} \exp\left(-\frac{|\mathbf{r}-\mathbf{r}_i|^2}{R_{q_i}^2}\right) \tag{S5}$$

$$|\mu_i| \rho_{\mu_i}(\mathbf{r}) = \frac{|\mu_i|}{\pi^{3/2} R_{\mu_i}^3} \hat{n}_i \cdot \nabla_{\mathbf{r}_i} \left[\exp\left(-\frac{|\mathbf{r}-\mathbf{r}_i|^2}{R_{\mu_i}^2}\right)\right] \tag{S6}$$

where R_{q_i} and R_{μ_i} are the Gaussian widths of charges and dipoles, respectively. $\hat{n}_i$ is the unit vector corresponding to the direction of the i-th dipole. The interaction kernels are then defined as:[1,4]

$$\begin{cases} T_{ij}^{qq} = \frac{1}{|\mathbf{r}_{ij}|} \text{erf}\left(\frac{|\mathbf{r}_{ij}|}{R_{q_i-q_j}}\right) \\ \boldsymbol{T}_{ij}^{q\mu} = \nabla_{r_i} T_{ij}^{qq} \\ \boldsymbol{T}_{ij}^{\mu\mu} = \nabla_{r_j} \boldsymbol{T}_{ij}^{q\mu} \end{cases} \tag{S7}$$

where $R_{q_i-q_j} = \sqrt{R_{q_i}^2 + R_{q_j}^2}$.

The equations 4 and 5 in the main text define the equations of motion for both charges and dipoles in the frequency domain. However, they can also be reformulated by exploiting the formalism recently developed by some of the present authors.[5] In particular, the Drude interaction matrix $\mathbf{K}$ can be defined as:

$$K_{ij} = (1-f(r_{ij}))K_{ij}^{\text{dru}} = \underbrace{\frac{2n_0}{1/\tau - \mathrm{i}\omega}}_{w(\omega)} \underbrace{(1-f(r_{ij}))\frac{\mathscr{A}_{ij}}{r_{ij}}}_{\overline{K}_{ij}} = w(\omega)\overline{K}_{ij}, \tag{S8}$$

where f is the Fermi damping function, w is a frequency-dependent complex scalar and $\overline{K}_{ij}$ is a real symmetric matrix. The diagonal elements are assumed to be zero, i.e. $K_{ii} = \overline{K}_{ii} = 0$ for $i = 1, \ldots, N$.

By plugging into the equations the partitioning of the Drude matrix reported in eq. S8 and dividing for w, we obtain for the ωFQ response:

$$\sum_{j=1}^{N}\left(\sum_{k=1}^{N}\overline{K}_{ik}(T_{kj}^{\text{qq}} - T_{ij}^{\text{qq}}) + \mathrm{i}\frac{\omega}{w(\omega)}\delta_{ij}\right)q_j + \sum_{j=1}^{N}\left(\sum_{k=1}^{N}\overline{K}_{ik}(T_{kj}^{\text{q}\mu} - T_{ij}^{\text{q}\mu})\right)\boldsymbol{\mu}_j = \sum_{k=1}^{N}\overline{K}_{ik}(V_i^{\text{ext}} - V_k^{\text{ext}}). \tag{S9}$$

We can now define the following shorthand notation

$$
\begin{aligned}
A^q_{ij} &= \sum_{k=1}^{N} \overline{K}_{ik}(T^{\mathrm{qq}}_{kj} - T^{\mathrm{qq}}_{ij}) = (\overline{K} - P)T^{\mathrm{qq}} \\
A^\mu_{ij} &= \sum_{k=1}^{N} \overline{K}_{ik}(T^{\mathrm{q}\mu}_{kj} - T^{\mathrm{q}\mu}_{ij}) = (\overline{K} - P)T^{\mathrm{q}\mu} \\
R_i &= \sum_{k=1}^{N} \overline{K}_{ik}(V^{\mathrm{ext}}_{i} - V^{\mathrm{ext}}_{k}) = -(\overline{K} - P)V \\
z(\omega) &= -\mathrm{i}\frac{\omega}{w(\omega)}, \qquad z'(\omega) = -\frac{1}{\alpha^\omega}.
\end{aligned}
$$

By this, the ωFQFμ linear system can be expressed as

$$
\left[\begin{pmatrix} A^q & A^\mu \\ T^{\mu \mathrm{q}} & T^{\mu\mu} \end{pmatrix} - \begin{pmatrix} z(\omega) I_N & 0 \\ 0 & z'(\omega) I_N \end{pmatrix}\right] \begin{pmatrix} \mathbf{q} \\ \boldsymbol{\mu} \end{pmatrix} = \begin{pmatrix} \mathbf{R} \\ -\mathbf{E}^{\mathrm{ext}} \end{pmatrix}.
$$

It can be seen that the left-hand side of the linear system is composed by a real frequency-independent nonsymmetric 2×2 block matrix with a shift on the diagonal for the complex scalar frequency-dependent quantities z, z'.

The total ωFQFμ matrix without the diagonal shift can also be written as

$$
\begin{pmatrix} A^q & A^\mu \\ T^{\mu \mathrm{q}} & T^{\mu\mu} \end{pmatrix} = \begin{pmatrix} (\overline{K} - P)T^{\mathrm{qq}} & (\overline{K} - P)T^{\mathrm{q}\mu} \\ T^{\mu \mathrm{q}} & T^{\mu\mu} \end{pmatrix} = \begin{pmatrix} (\overline{K} - P) & 0 \\ 0 & I \end{pmatrix} \begin{pmatrix} T^{\mathrm{qq}} & T^{\mathrm{q}\mu} \\ T^{\mu \mathrm{q}} & T^{\mu\mu} \end{pmatrix}
$$

showing its relationship with respect to the FQFμ interaction kernel.[1] Once charges and dipoles are calculated, it is possible to compute the complex dipole $\bar{\xi}$ as:

$$
\bar{\xi}(\omega) = \sum_i q_i(\omega)\mathbf{r}_i + \sum_i \boldsymbol{\mu}_i(\omega) \tag{S10}
$$

The complex polarizability of the whole system $\bar{\alpha}$ then reads:

$$
\bar{\alpha}(\omega)_{\alpha\beta} = \frac{\bar{\xi}_\alpha}{E_{0,\beta}} \tag{S11}
$$

where, α, β are x,y,z directions. Finally, the absorption cross section $\sigma(\omega)$ can be computed:

$$\sigma_i(\omega) = \frac{4\pi\omega}{3c}\mathrm{Tr}(\bar{\alpha}) \tag{S12}$$

The induced electric field at a generic point $\mathbf{r_j}$ can finally be calculated as:

$$\begin{aligned}\mathbf{E}_X(\mathbf{r}_j) = &-\sum_i^{N_{atoms}} q_i^X \left[\frac{\mathbf{r}_{ij}}{|\mathbf{r}_{ij}|^3}\left(\mathrm{erf}\left(\frac{|\mathbf{r}_{ij}|}{R_{q_i}}\right) - \frac{2\,|\mathbf{r}_{ij}|}{\sqrt{\pi}R_{q_i}}\exp\left(-\frac{|\mathbf{r}_{ij}|^2}{R_{q_i}^2}\right)\right)\right] \\ &- \mu_i^X \left(\frac{3\mathbf{r}_{ij}\otimes\mathbf{r}_{ij} - |\mathbf{r}_{ij}|^2\mathbf{I}}{|\mathbf{r}_{ij}|^5}\left[\mathrm{erf}\left(\frac{|\mathbf{r}_{ij}|}{R_{\mu_i}}\right) - \frac{2}{\sqrt{\pi}}\frac{|\mathbf{r}_{ij}|}{R_{\mu_i}}\exp\left(-\frac{|\mathbf{r}_{ij}|^2}{R_{\mu_i}^2}\right)\right]\right. \\ &\left. - \frac{4}{\sqrt{\pi}R_{\mu_i}^3}\frac{\mathbf{r}_{ij}\otimes\mathbf{r}_{ij}}{|\mathbf{r}_{ij}|^2}\exp\left(-\frac{|\mathbf{r}_{ij}|^2}{R_{\mu_i}^2}\right)\right)\end{aligned} \tag{S13}$$

where $\mathbf{r}_{ij}$ is the vector connecting atom i and point j, whereas X indicates real (Re) and imaginary (Im) parts of both the electric field and ωFQs. The enhancement factor at point $\mathbf{r}_j$ is then obtained as:

$$\Upsilon(\mathbf{r}_j) = \frac{|E|}{E_0} = \frac{\sqrt{|\mathbf{E}_{Re}(\mathbf{r}_j)|^2 + |\mathbf{E}_{Im}(\mathbf{r}_j)|^2}}{|\mathbf{E}_0|} \tag{S14}$$

where E_0 is the amplitude of the incident electric field.

S1.1 Parametrization

S1.1.1 Static parameters

ωFQFμ static response is defined in terms of η, α^0, R_q and R_μ parameters, the latters defining the interaction kernels T^{qq}, $T^{q\mu}$ and $T^{\mu\mu}$. To find their best values, we fit our model on static polarazibilities of eight/seven small Ag/Au clusters reported in Ref. 6, where reference static polarizabilities calculated at the DFT level were provided.

S1.1.2 Dynamic parameters

As stated above, the interband effects are taken into account by the dynamic atomic polarizabilities α_i^ω. To find an expression for α^ω, we recall that the dielectric function of a bulk material can be divided into two

Table S1: Static Polarizabilities for small silver and gold clusters as calculated by ωFQFμ and reference DFT values reproduced from Ref. 6.

Structure	ωFQFμ	DFT
Ag_4	193.94	168.33
Ag_6	307.89	256.86
Ag_8	350.96	317.59
Ag_{20}	777.46	765.48
Ag_{34}	1229.09	1186.24
Ag_{40}	1470.73	1431.23
Ag_{58}	2027.71	1968.87
Ag_{68}	2374.35	2369.59

Structure	ωFQFμ	DFT
Au_4	150.52	146.54
Au_6	236.81	222.14
Au_8	336.89	305.89
Au_{20}	410.08	418.92
Au_{34}	735.57	742.18
Au_{40}	1110.39	1095.04
Au_{58}	1972.23	1896.91

parts: the purely conductive Drude expression ε_D and the interband contribution ε_{IB}:[7–9]

$$\varepsilon(\omega) = \varepsilon_D(\omega) + \varepsilon_{IB}(\omega) \tag{S15}$$

$$\varepsilon_D(\omega) = 1 - \frac{\omega_p^2}{\omega^2 + i\gamma\omega} \tag{S16}$$

where γ is a damping factor and ω_p is the plasma frequency of the metal. Notice that these parameters define the matrix elements K_{ij} in Eq. 1 (main text) through the following relations:

$$\begin{cases} \gamma = 1/\tau \\ n_0 = \sigma_0/\tau \\ \omega_p^2 = 4\pi n_0 = 4\pi\sigma_0/\tau \end{cases} \tag{S17}$$

where σ_0 is the static conductivity. In ωFQFμ, the macroscopic polarization $\mathbf{P}$ is determined by both charges and dipoles, and from classical electrodynamics[8,10] it can be expressed as:

$$\mathbf{P} = \frac{\varepsilon - 1}{4\pi}\mathbf{E} = \mathbf{P}_D + \mathbf{P}_{IB} = \frac{\varepsilon_D - 1}{4\pi}\mathbf{E} + n\alpha^{\omega}\mathbf{E}$$

where, n is the number density of atoms. The atomic polarizability due to interband transitions (α^{ω}) is then obtained as a function of the interband dielectric constant:

$$\alpha^{\omega} = \frac{\varepsilon_{IB}}{4\pi n} \tag{S18}$$

The dynamic polarizability α^{ω} finally enters in Eq. 3 (main text), which is therefore a complex-value parameter whose imaginary part is connected to interband absorption. Note that α^{ω} in Eq. S18 is obtained without applying local field corrections, because it provides better results when used in ωFQFμ calculations.

In this work, the dynamical ωFQFμ parameters, i.e. Drude and interband parameters, are obtained from the dielectric function of Etchegoin et al.[11] fitted by Johnson and Christy[12,13] for both Silver and Gold. We can recover α^{ω} by exploiting Eq. S18. Ag and Au interband complex polarizabilities exploited in this work are reported in Figs. S1

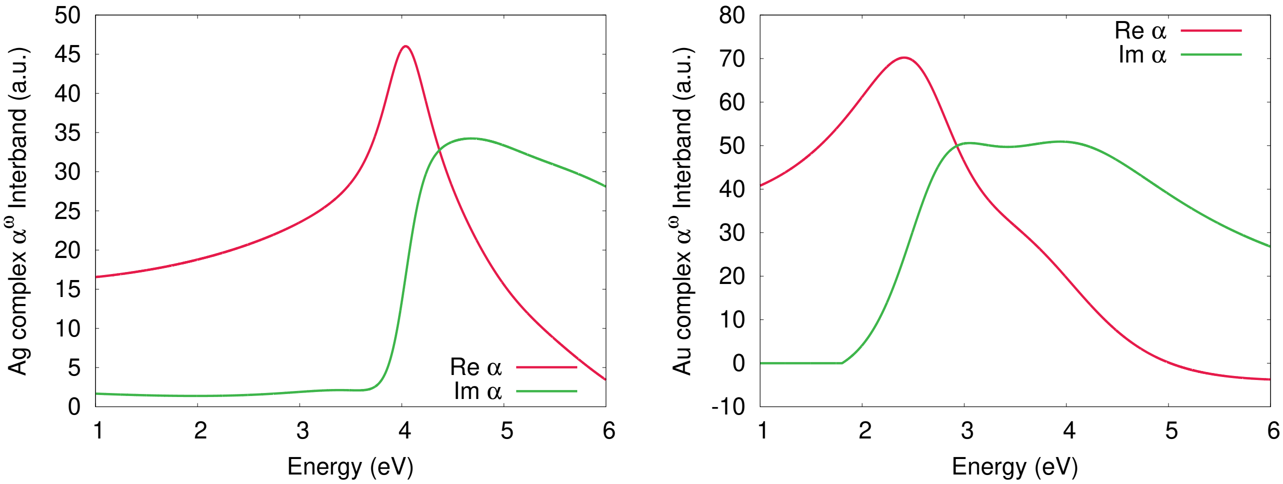

Figure S1: Ag and Au α^{ω} obtained from the interband Etchegoin dielectric function. Real (Re) and imaginary (Im) parts of the polarizability are given in atomic units (a.u.).

$\mathbf{A}^{qq}$, $\mathbf{A}^{q\mu}$ and $\mathbf{f}^{q}$ are defined in terms of the matrix K_{ij}, which is introduced in Eq. 1 (main text). Such matrix is constituted by a Drude contribution, which depends on three parameters: the static conductivity σ_0, the scattering time τ, and the effective area A_{ij}. K_{ij} also contains the Fermi $1 - f(l_{ij})$ factor (see Eq. 1), which depends on three parameters, i.e. l_{ij}^{0}, d and s.

Among these six parameters, the first neighbours distance l_{ij}^{0} is fixed by the material that composes the NP. σ_0 and τ can be recovered from the literature for most metals, or from the Drude part of the permittivity

function ε (see Eq. S16). It is worth noticing that σ_0 and τ define the plasma frequency of the material through the relation $\omega_p^2 = 4\pi\frac{\sigma_0}{\tau}$, which can be obtained from the experimental permittivity function.

A_{ij}, d and s are instead determined by fitting their value so that ωFQFμ reproduces the plasmon resonance frequencies for small silver nanorods, for which reference ab-initio values are reported in the literature. We selected silver nanorods because their PRFs are far away from the interband transitions, and therefore they can be reproduced by a simple Drude model. The results obtained at the ωFQFμ level and their reference counterparts are reported in Tabs. S2.

Table S2: Absorption frequency (eV) of Ag nanorods predicted by $\omega FQF\mu$ and compared to ab-initio TDDFT results taken from ref. 14.

Nanorod	$\omega FQF\mu$ (eV)	TDDFT (eV)
Ag_{37}	2.60	2.58
Ag_{67}	1.93	1.93
Ag_{103}	1.48	1.49
Ag_{145}	1.18	1.21

Nanorod	$\omega FQF\mu$ (eV)	TDDFT (eV)
Au_{37}	2.07	2.32
Au_{67}	1.65	1.81
Au_{103}	1.33	1.40
Au_{145}	1.08	1.14

We finally summarize the parameters exploited in the present work in Tabs. S3 and S4. It is worth noting that the values of A_{ij} (=R_i^2 in ωFQFμ) and α^0 parameters are almost in perfect agreement with reference data.[15–17] In particular:

- Ag $R_i = 1.64$ Å (in good agreement with the calculated Ag radius[16]).
- Ag $\alpha^0 = 49.9843$ a.u. (in perfect agreement with Ref. 6).
- Au $R_i = 1.64$ Å (in good agreement with Au van der Waals radius.[18])
- Au $\alpha^0 = 39.5297$ a.u. (in perfect agreement with Ref. 6.)

Table S3: $\omega FQF\mu$ parameters for Silver used in calculations. t.w.: this work.

Parameter	Value (a.u.)	Value (common units)	Equation	Reference
τ	1633.608	39.52 fs	1	13
ω_p	0.330	8.99 eV	1 - S17	13
s	1.1	1.1	2	t.w.
d	12	12	2	t.w.
A_{ij}	9.61	2.69 Å^2	1	t.w.
l_{ij}^0	5.45	2.88 Å	2	17
n	0.00867	0.097 mol/cm^3	S18	17
α^0	49.9843	7.4069 Å^3	S3	t.w.
η	0.4829	13.14 eV	S4	t.w.
R_q	3.378	1.788 Å	S5	t.w.
R_μ	2.369	1.254 Å	S6	t.w.

Table S4: $\omega FQF\mu$ parameters for Gold used in calculations. t.w.: this work.

Parameter	Value (a.u.)	Value (common units)	Equation	Reference
τ	318.018	7.69 fs	1	13
ω_p	0.319	8.67 eV	1 - S17	13
s	1.1	1.1	2	t.w.
d	12	12	2	t.w.
A_{ij}	9.61	2.69 Å^2	1	t.w.
l_{ij}^0	5.45	2.88 Å	2	17
n	0.00875	0.098 mol/cm^3	S18	17
α^0	39.5297	5.8577 Å^3	S3	t.w.
η	0.5268	14.33 eV	S4	t.w.
R_q	3.500	1.852 Å	S5	t.w.
R_μ	3.071	1.625 Å	S6	t.w.

S2 Computational details

ωFQFμ equations have been implemented in a stand-alone fortran95 code called nanoFQ. The final set of linear equations (Eq. 6 in the main text) is solved by a LU decomposition of the left hand side matrix for each input frequency, as explained in ref. 19. For $N_{atoms} > 10000$, the equations are solved by exploiting the GMRES algorithm (see Ref. 5), by imposing a Root Mean Square Error (RMSE) equal to 10^{-5}. The plasmon densities are then calculated through Eq. S5 and S6. All Ag/Au NP structures have been constructed by using an open source engine for molecular dynamics,[20] by imposing the lattice constant to be 4.08 Å in a Face-Centered Cubic (FCC) packing.[20,21]

S3 Results

S3.1 Model validation

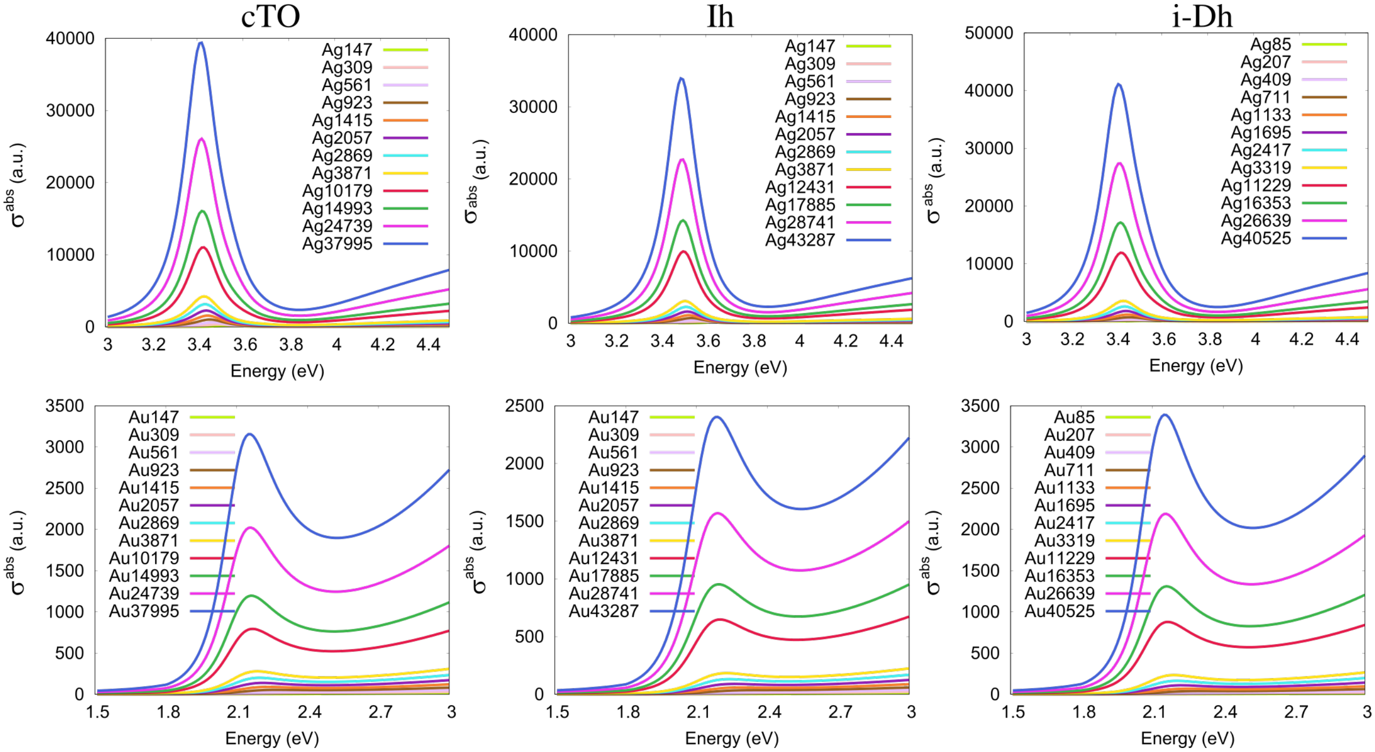

Figure S2: Ag and Au cTO, Ih, i-Dh NPs absorption cross section as a function of the number of atoms

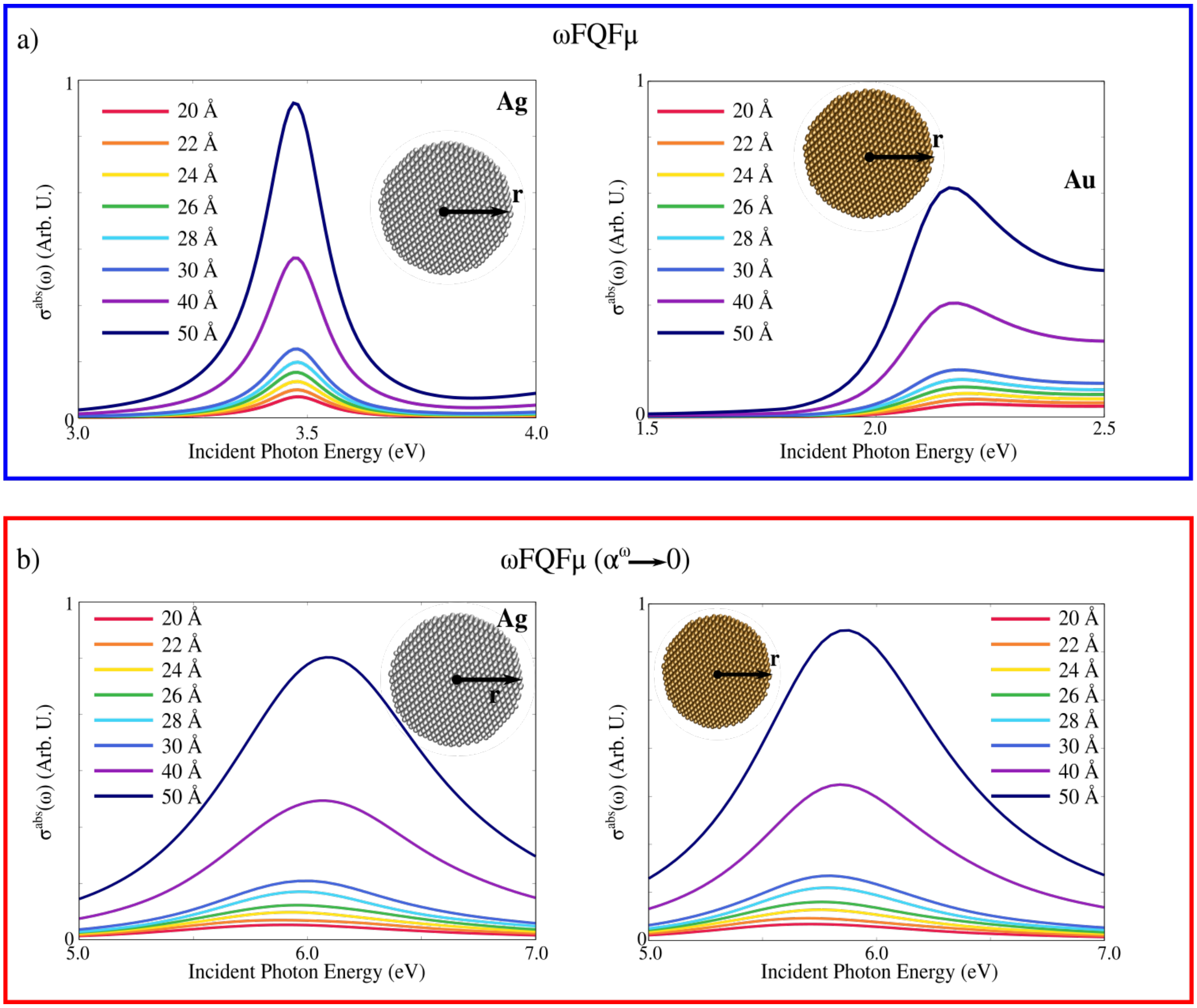

Figure S3: (a) ωFQFμ and (b) ωFQF$\mu(\alpha^{\omega} \rightarrow 0)$ σ^{abs} of spherical Ag and Au NPs as a function of the radius r (from 20 to 50 Å).

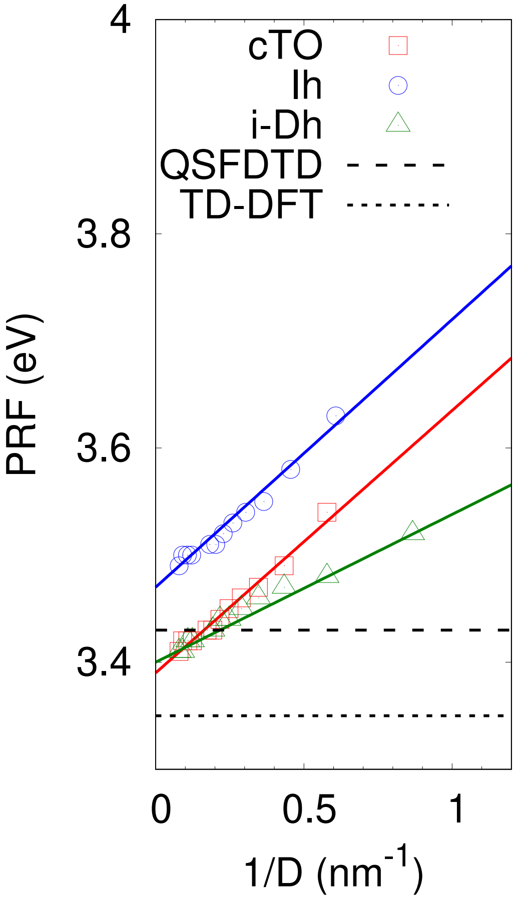

Figure S4: Ag cTO, Ih and i-Dh PRFs as a function of the inverse of the NP diameter. Reference TD-DFT and QSFDTD values at infinite distance reproduced from Ref. 22 are also reported.

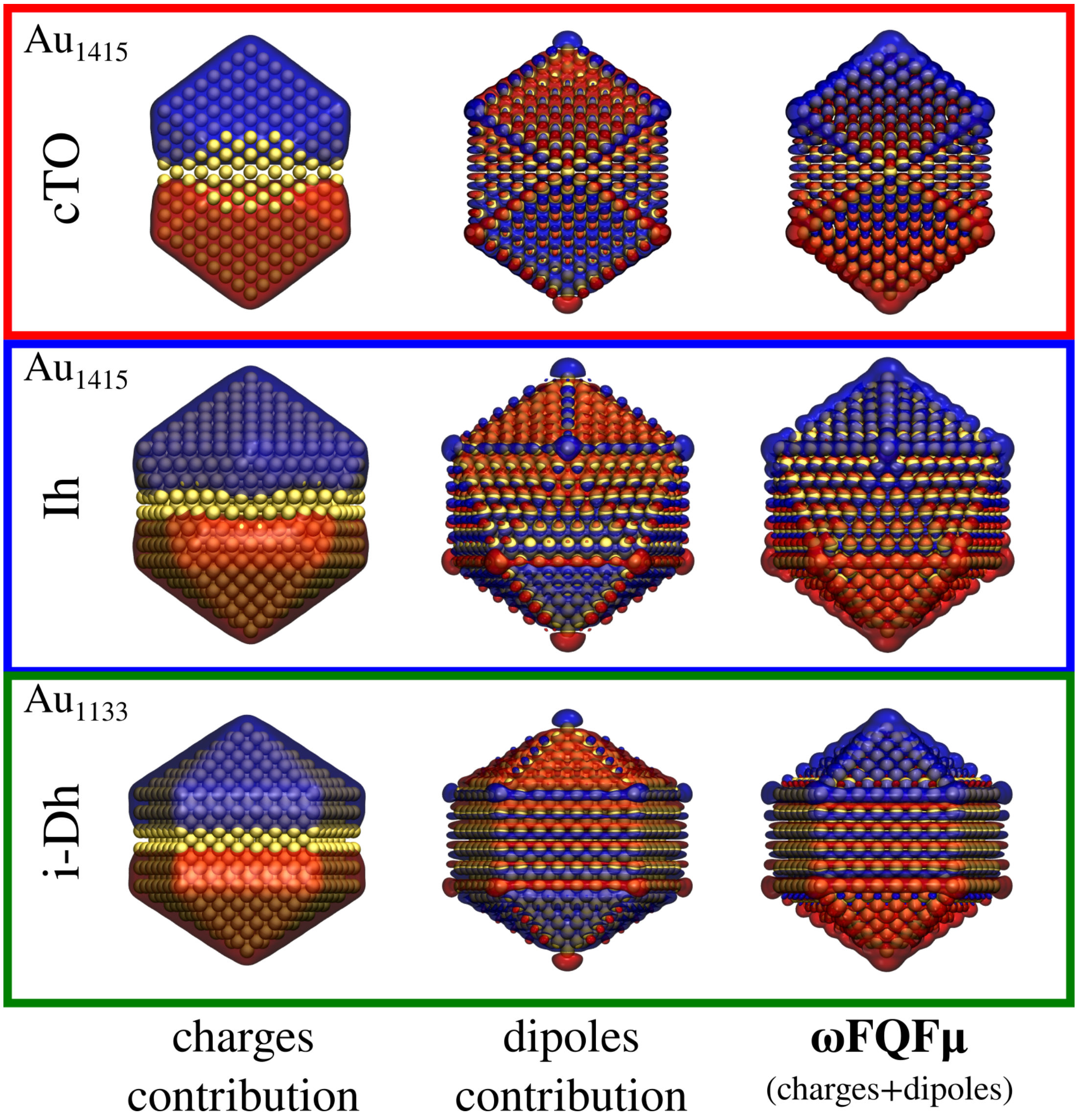

Figure S5: Au_{1415} cTO and Ih and Au_{1131} i-Dh charge and dipole contributions to the plasmon densities computed at the PRFs. The total ωFQFμ plasmon densities are also plotted. Density isovalue: 0.005 a.u.

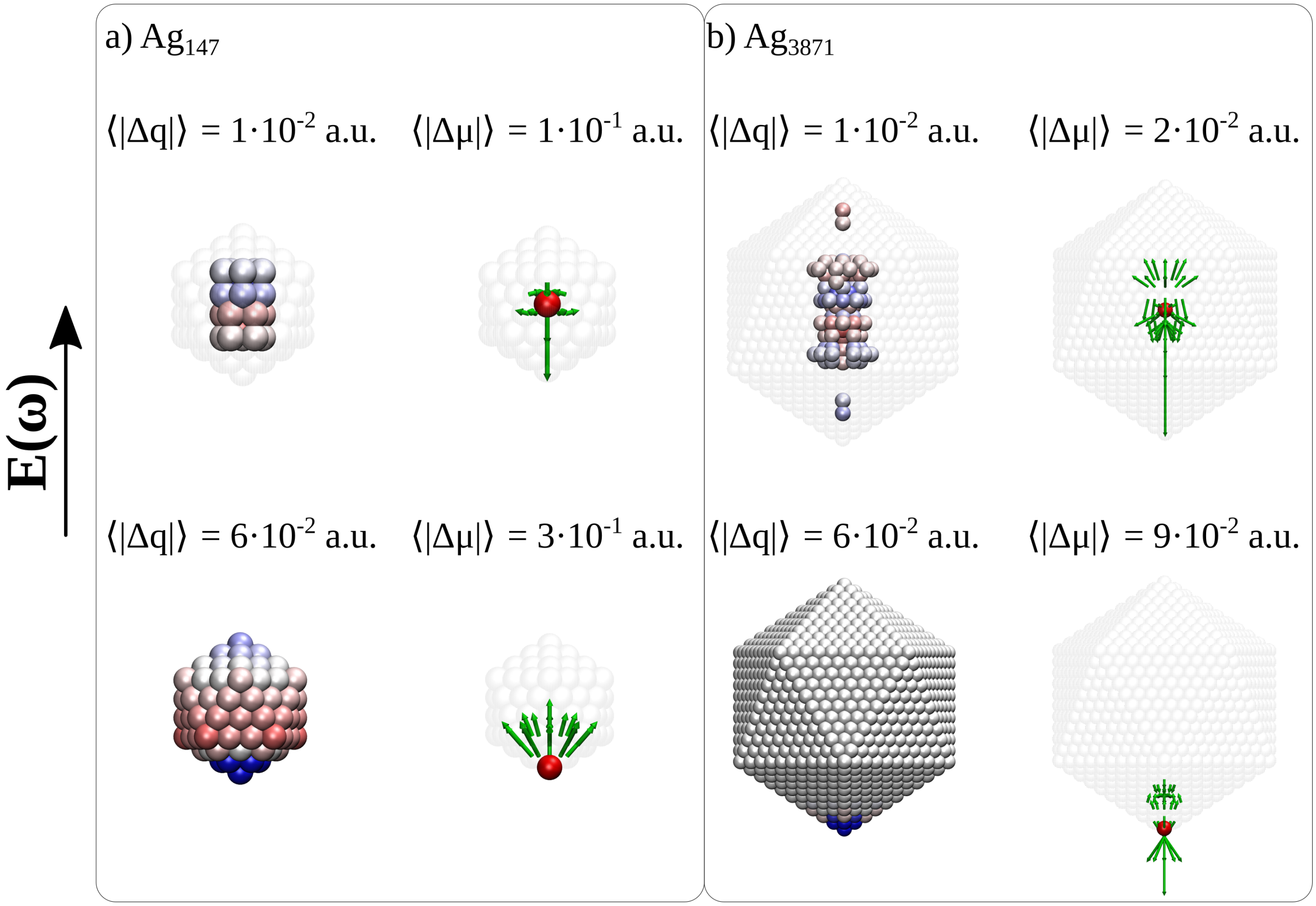

Figure S6: Graphical depiction of ωFQFμ non-local effects in Ag_{147} (a) and Ag_{3871} NPs (b). The perturbed atom is displayed in red and it is located at the surface (bottom) or in the middle (top) of the structure. The total electric potential and field acting on the selected atom is scaled by a factor 1.1. In both panels a and b, the differences between the unperturbed and the perturbed charges (left) and dipoles (right) are displayed. The average absolute deviations are also given. Clearly, the perturbation acting on a single atom non-locally perturbs other atoms of the structure.

S4 Subnanometer junctions

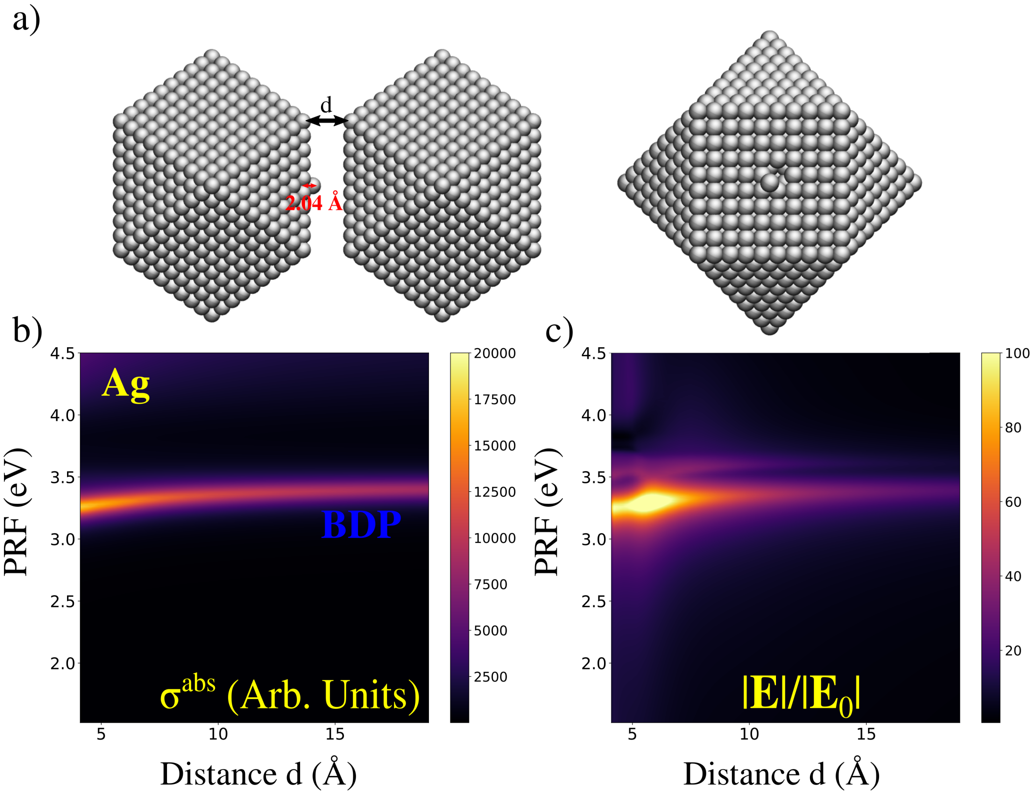

Figure S7: (a) Graphical depiction of surface roughness as derived from an atomic adjustment on the surface of one of the two Ag cTO NPs in the surface-surface arrangement (see right panel). The structure has been created by moving an Ag atom from its pristine atomic layer on the NP (100) surface to the next atomic layer at the center of the NP (100) surface (see right panel). The distance between the adatom and its original atomic layer is 2.04 Å, according to the Ag lattice constant. (b) Absorption cross section (left) and electric field enhancement (right) as a function of the distance between the two NPs. The distance between the two NPs is computed as in the pristine case (see Sec. 3.2 in the main text, and panel a, left). The electric field enhancement maps report the $|\mathbf{E}|/|\mathbf{E}_0|$ factors computed at the center of the plane at $d/2$.

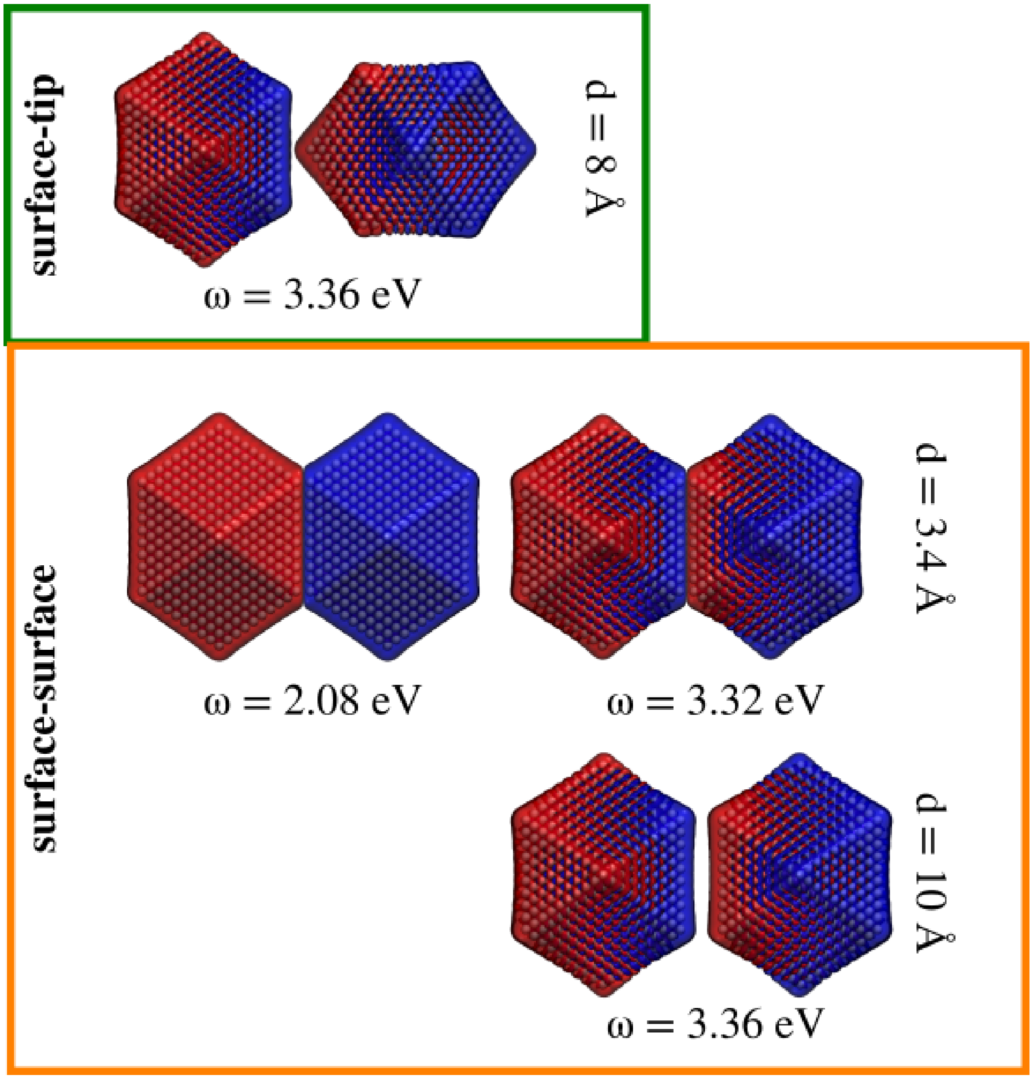

Figure S8: Main plasmon densities for Ag cTO_{2869} dimers in surface-tip (top) and surface-surface (bottom) geometrical arrangements. Density isovalue: 0.002 a.u.

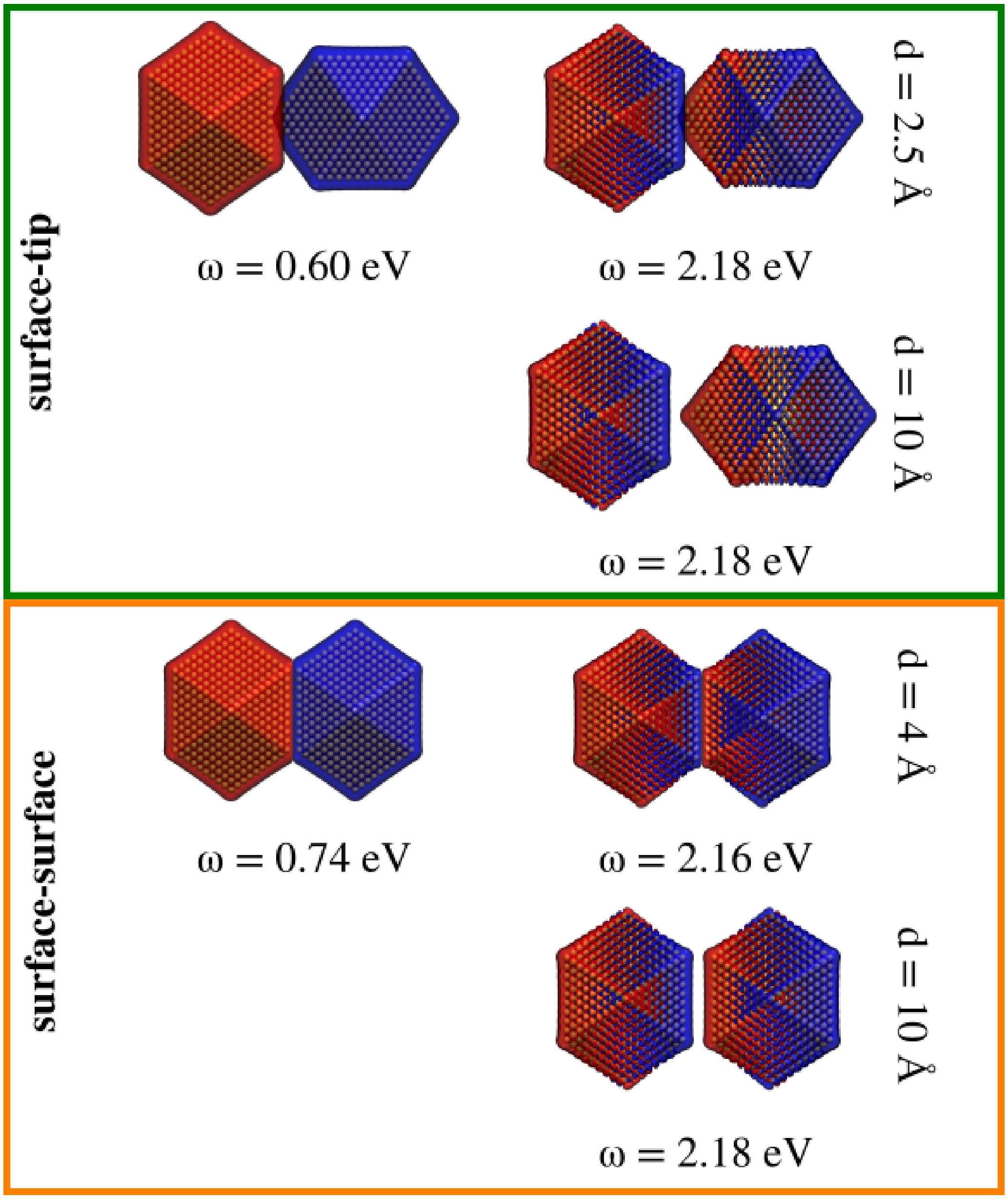

Figure S9: Main plasmon densities for Au cTO_{2869} dimers in surface-tip (top) and surface-surface (bottom) geometrical arrangements. Density isovalue: 0.005 a.u.

References

(1) Giovannini, T.; Puglisi, A.; Ambrosetti, M.; Cappelli, C. Polarizable QM/MM approach with fluctuating charges and fluctuating dipoles: the QM/FQFμ model. *J. Chem. Theory Comput.* **2019**, *15*, 2233–2245.

(2) Giovannini, T.; Riso, R. R.; Ambrosetti, M.; Puglisi, A.; Cappelli, C. Electronic transitions for a fully polarizable qm/mm approach based on fluctuating charges and fluctuating dipoles: linear and corrected linear response regimes. *J. Chem. Phys.* **2019**, *151*, 174104.

(3) Giovannini, T.; Grazioli, L.; Ambrosetti, M.; Cappelli, C. Calculation of ir spectra with a fully polarizable qm/mm approach based on fluctuating charges and fluctuating dipoles. *J. Chem. Theory Comput.* **2019**, *15*, 5495–5507.

(4) Mayer, A. Formulation in terms of normalized propagators of a charge-dipole model enabling the calculation of the polarization properties of fullerenes and carbon nanotubes. *Phys. Rev. B* **2007**, *75*, 045407.

(5) Lafiosca, P.; Giovannini, T.; Benzi, M.; Cappelli, C. Going Beyond the Limits of Classical Atomistic Modeling of Plasmonic Nanostructures. *J. Phys. Chem. C* **2021**, *125*, 23848–23863.

(6) Jensen, L. L.; Jensen, L. Electrostatic interaction model for the calculation of the polarizability of large noble metal nanoclusters. *J. Phys. Chem. C* **2008**, *112*, 15697–15703.

(7) Pelton, M.; Bryant, G. W. *Introduction to metal-nanoparticle plasmonics*; John Wiley & Sons, 2013; Vol. 5.

(8) Jackson, J. D. *Classical electrodynamics*; John Wiley & Sons, 2007.

(9) Trügler, A. *Optical properties of metallic nanoparticles*; Springer, 2011.

(10) Landau, L. D.; Bell, J.; Kearsley, M.; Pitaevskii, L.; Lifshitz, E.; Sykes, J. *Electrodynamics of continuous media*; elsevier, 2013; Vol. 8.

(11) Etchegoin, P. G.; Le Ru, E.; Meyer, M. An analytic model for the optical properties of gold. *J. Chem. Phys.* **2006**, *125*, 164705.

(12) Blaber, M. G.; Henry, A.-I.; Bingham, J. M.; Schatz, G. C.; Van Duyne, R. P. LSPR Imaging of Silver Triangular Nanoprisms: Correlating Scattering with Structure Using Electrodynamics for Plasmon Lifetime Analysis. *The Journal of Physical Chemistry C* **2012**, *116*, 393–403.

(13) Johnson, P. B.; Christy, R.-W. Optical constants of the noble metals. *Phys. Rev. B* **1972**, *6*, 4370.

(14) Sinha-Roy, R.; Garcia-Gonzalez, P.; Weissker, H.-C.; Rabilloud, F.; Fernandez-Dominguez, A. I. Classical and ab Initio Plasmonics Meet at Sub-nanometric Noble Metal Rods. *ACS Photonics* **2017**, *4*, 1484–1493.

(15) Naserifar, S.; Brooks, D. J.; Goddard III, W. A.; Cvicek, V. Polarizable charge equilibration model for predicting accurate electrostatic interactions in molecules and solids. *J. Chem. Phys.* **2017**, *146*, 124117.

(16) Clementi, E.; Raimondi, D.; Reinhardt, W. P. Atomic screening constants from SCF functions. II. Atoms with 37 to 86 electrons. *J. Chem. Phys.* **1967**, *47*, 1300–1307.

(17) Haynes, W. *CRC Handbook of Chemistry and Physics*; CRC Press: Boca Raton, 2014.

(18) Mantina, M.; Chamberlin, A. C.; Valero, R.; Cramer, C. J.; Truhlar, D. G. Consistent van der Waals radii for the whole main group. *J. Phys. Chem. A* **2009**, *113*, 5806–5812.

(19) Giovannini, T.; Rosa, M.; Corni, S.; Cappelli, C. A classical picture of subnanometer junctions: an atomistic Drude approach to nanoplasmonics. *Nanoscale* **2019**, *11*, 6004–6015.

(20) Gezelter, J.; Kuang, S.; Marr, J.; Stocker, K.; Li, C.; Vardeman, C.; Lin, T.; Fennell, C.; Sun, X.; Daily, K., et al. OpenMD, an open source engine for molecular dynamics. *University of Notre Dame, Notre Dame, IN* **2010**,

(21) Bonatti, L.; Gil, G.; Giovannini, T.; Corni, S.; Cappelli, C. Plasmonic Resonances of Metal Nanoparticles: Atomistic vs. Continuum Approaches. *Front. Chem.* **2020**, *8*, 340.

(22) Kuisma, M.; Sakko, A.; Rossi, T. P.; Larsen, A. H.; Enkovaara, J.; Lehtovaara, L.; Rantala, T. T. Localized surface plasmon resonance in silver nanoparticles: Atomistic first-principles time-dependent density-functional theory calculations. *Phys. Rev. B* **2015**, *91*, 115431.
